# Accuracy of Seismic Response Evaluation of Two-Dimensional Analysis Model with Rigid Joints for RC Frame Buildings

**DOI:** 10.3390/ma15228027

**Published:** 2022-11-14

**Authors:** Jae-Do Kang, Takuya Nagae, Seong-Hoon Jeong, Koichi Kajiwara

**Affiliations:** 1 Earthquake Disaster Mitigation Center, Seoul Institute of Technology, Seoul 03909, Korea; 2 National Research Institute for Earth Science and Disaster Resilience, Miki 673-0515, Japan; 3 Disaster Mitigation Research Center, Nagoya University, Nagoya 464-8601, Japan; 4 Department of Architectural Engineering, Inha University, Incheon 22212, Korea; 5 Earthquake Disaster Mitigation Research Division, National Research Institute for Earth Science and Disaster Resilience, Miki 673-0515, Japan

**Keywords:** effective slab width, beam, two-dimensional numerical model, shaking-table test

## Abstract

Three- or two-dimensional (2D) numerical models are used for the evaluation of the seismic performance of reinforced concrete (RC) buildings. This study examines a 2D numerical model for a specimen used in a full-scale four-story RC shaking-table test and evaluates the accuracy of the seismic response of the 2D numerical model, which is composed of a square fiber section model for the columns, a T-shape fiber section model for the beam and slab, and a rigid joint model for the beam–column joint. A parametric analysis of the effective slab width is performed to analyze its effects on the modal shape and natural period. The results suggest that the primary natural period of the considered model is almost identical to that associated with the experimental results. The applicability of the 2D numerical model for estimating the seismic response of the structure is established. By comparing the results of the seismic analysis and the experiment in the 50% amplitude of the JMA-Kobe wave, which corresponds to slightly exceeding VII on the modified Mercalli intensity scale, the root-mean-square percentage error of the 2D numerical model is 1.03% for the floor acceleration and 4.7% for the inter-story drift. Thus, the analytical model used in this study has sufficient accuracy in evaluating the seismic performance of buildings constructed in regions with a maximum seismic intensity of VII.

## 1. Introduction

According to references [1,2], structural systems that can withstand seismic excitations are classified as follows: (1) moment-resisting frames, (2) building frames, (3) bearing walls, (4) dual types with special moment frames capable of resisting at least 25% of the prescribed seismic forces, and (5) dual types with intermediate moment frames capable of resisting at least 25% of the prescribed seismic forces. The special moment-resisting frame (SMRF), which is a type of moment-resisting frame system, and special shear wall systems, which are types of bearing wall systems, are often used in regions of strong seismicity (e.g., Japan, United States). In regions of low and moderate seismicity, the intermediate moment-resisting frame (IMRF), which is also one of the moment-resisting frame systems, has been used extensively. American standards [1] distinguish between the SMRF and IMRF according to the reinforcement bar arrangements, but Japanese building standards do not. However, the details of the SMRF in Japan are similar to the American standards. The design criteria of reinforced concrete (RC) moment-resisting frames include the cross-sectional area, column transverse reinforcement spacing, and the beams’ flexural strength. In general, due to effects such as the (1) slab, (2) axial beam force, (3) external force distribution, and (4) strain hardening, the ultimate flexural strength of the beams of the RC building during an earthquake is larger than the ultimate strength of a single beam [3]. To consider these according to the design guidelines of the Architectural Institute of Japan (AIJ) for earthquake-resistant reinforced concrete buildings based on the ultimate strength concept [4], the upper limit of the beam flexural strength is set to become the design purpose [3]. Hence, it is important to accurately evaluate the effective width of the slab in structural calculations. In allowable stress calculations [5], approximately 1/10 of the span is considered an effective slab width (one side). Using static loading tests of the three-dimensional (3D) partial frame, evaluations of the effect of the effective slab width on the flexural strength of the beams were conducted by Kabeyasawa et al. [3], who clarified the mechanism based on which the slab reinforcements at the top and bottom of almost the entire width could effectively work on the flexural strength of the beams. However, the effect of effective slab width during strong earthquake motion has not been evaluated using the results of a shaking-table test.

Meanwhile, the experimental reports on the seismic performance of existing RC frame buildings, which were designed before the introduction of seismic codes in countries with strong seismicity, emphasized damage to the joint panel zone [6,7,8,9,10]. Owing to the necessity for the evaluation of the seismic performance of existing RC buildings, the accuracy of the two-dimensional (2D) models for RC beam–column joints have been developed and investigated, including the (1) rigid joint, (2) rotational hinge joint [11,12], and (3) constitutive model, which consists of either rigid elements and rotational springs, bar-slip springs, or axis springs [13,14,15,16,17,18]. The first 2D macro-element model for beam–column joints was proposed by Tajiri and Shiohara [19] based on the developed mechanical model, which captured the nonlinear behavior of RC beam–column joints [20]. By extending the 2D macro-element model to beam–column joints, a 3D macro-element model has been proposed [21]. The macro-element models are an effective tool to evaluate the seismic performance of buildings’ ductile RC frames in strong seismicity regions. To evaluate the accuracy and ease of use of existing 2D models for RC beam–column joints, modeling techniques and constitutive behavior are assessed based on maximum strength comparisons [22]. According to reference [22], the maximum strength difference between the used models, such as the rigid joint and three rotational hinge joint models, is 13%. In other words, the rigid joint model is also used for the evaluation of the maximum strength of buildings, except for buildings with non-seismic joints. However, in that study, the effect of the joint model on seismic performance, such as inter-story drift, is not evaluated. Therefore, it is necessary to evaluate the accuracy of the displacement response estimation according to the joint. In addition, in order to evaluate seismic performance using numerical models of the member, which have been developed by laboratory experiments, including the joint during earthquakes, it is necessary to evaluate by comparing it with the result of the shaking-table test.

Experiments using a full-scale earthquake testing facility, commonly referred to as “E-Defense”, have been conducted since 2005 to elucidate the collapse process of buildings and improve seismic performance [23]. Among these, the test specimen of the full-scale experiment conducted in 2010 [24] was a four-story RC building with a rectangular plane. Its structural system was a moment-resisting frame in the long-side direction and a multistory shear wall in the short-side direction. By gradually increasing the input seismic motion, the maximum inter-story drift ratios reached approximately 0.045 rad (4.5%) in the long-side direction and approximately 0.055 rad (5.5%) in the short-side direction [25]. Tuna et al. [26] constructed a numerical model that represented walls with a fiber model and compared their seismic performance along the short-side direction. Liu et al. [27] constructed a 3D numerical model composed of an MVLEM model, which placed rigid beams at the upper and lower floor levels and used shear springs and multiple vertical springs in the center for RC shear walls, and a fiber section model for columns and beams. Kang et al. [28] constructed a 2D numerical model that consisted of columns, beams, and walls represented by a fiber model; in this model, each frame was connected by rigid truss elements. In this paper [28], the effect of effective slab width on stiffness was evaluated. However, the seismic response using the 2D numerical model has not been conducted. With the development of computer technology, the numerical model has also developed and involves a complex mechanical model to express the nonlinear behavior of members. Recent models based on few parameters having clear mechanical significance [29,30] may be adopted to address such an issue. Although these technologies are useful for evaluating the seismic performance of buildings in regions of strong seismicity, their applicability may be low in evaluating buildings in regions of low and moderate seismicity because these technologies require expert interpretation and knowledge about seismic engineering. The experiment conducted by Nagae et al. [24,25] enabled the evaluation of the model’s accuracy depending on the level of seismic intensity because the input seismic motion was gradually increased. Therefore, it is possible to examine the applicable model according to the expected seismic intensity.

The objective of this study is (1) the construction of a 2D model, which is often used in performance design, and (2) the evaluation of the accuracy of that model. To analyze the effective slab width, we use a 2D numerical model composed of a square fiber section model for the columns, a T-shape fiber section model for the beam and slab, and a rigid joint model for the beam–column joint. It is important to accurately evaluate the natural period and mode shape in the response evaluations of structures. Therefore, we conduct a parametric analysis of the slab width to analyze the effects of the slab width on the modal shape and natural period of the model. Based on elastic parametric analysis results, the beam and slab are modeled by a T-shape beam to consider the effective slab width of 450 mm. Using a 2D numerical model in which the joint is modeled in the rigid region, we quantitatively evaluate the accuracy of the seismic performance of the used model. In addition, the accuracy of the model used in this study is evaluated by classifying it into two kinds of seismic intensity.

## 2. Building Specimen

### 2.1. Basic Plan and Design Criteria

**Figure 1:**
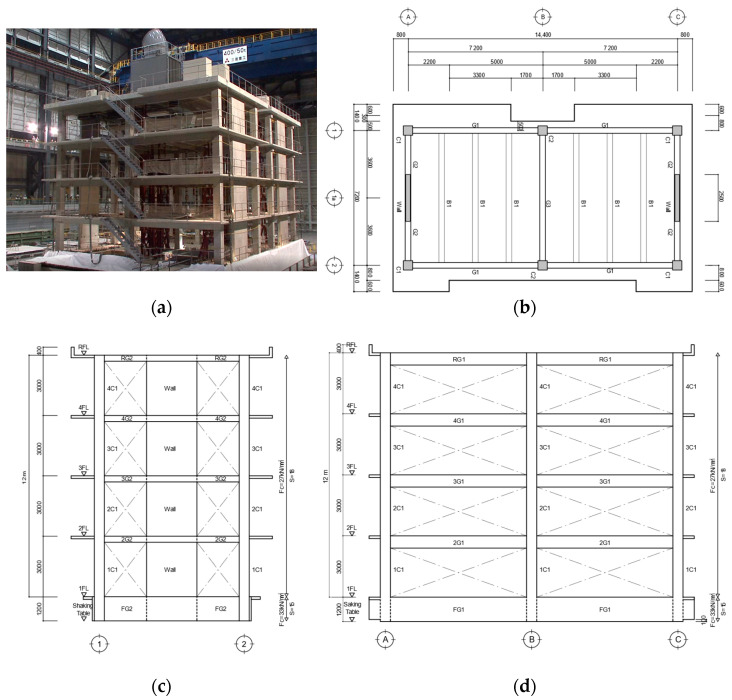
Configuration of test specimen and labeling of structural elements (unit: mm): (**a**) Photo of specimen installation on shake table (front: RC specimen; back: PT specimen); (**b**) plan of standard floor (second to fourth floors); (**c**) elevations of A and C frames (the shear wall frame); and (**d**) elevations of first and second frames (the moment-resisting frame).

Figure 1a shows a photo of the specimen installation on the shake table. On the shaking table, an RC building and a post-tensioned (PT) building with members of the same size were fixed. The RC specimen was a four-story RC building with a rectangular plane and was composed of full-scale members. The height of the foundation was 1.2 m, and the distances of the floor heights from the first to the fourth floors were 3 m. The long-side direction, hereafter X-direction, was a moment-resisting frame. The short-side direction, hereafter Y-direction, had a multistory shear wall in the center of the exterior. The X-direction consisted of two spans equal to 7.2 m, and the Y-direction consisted of one span equal to 7.2 m. The building specimen was designed based on the 2010 version of the AIJ Standard for the structural calculation of RC structures [5] and the 2007 version of the guidelines for the technical standards of building structures [29]. The main design criteria were the following: (1) the allowable stress design was performed using the long-term and short-term loads, (2) the ratio of flexural strength between the column and beam (column–beam strength ratio) was approximately equal to one, and (3) when checking the lateral load-carrying capacity, the base shear force coefficient was in the range of 0.3–0.4. However, some beam–column joints did not meet the design guidelines [31] based on the AIJ Standard [32] because there were existing beam–column joints that did not satisfy these conditions. Based on the AIJ Standard for the structural calculation of RC structures [5], shear walls are a rectangular shape, not a barbell shape.

### 2.2. Design Overview

Figure 1 shows the photo, the floor plan, and the elevations of the specimen. Table 1 lists the weight of each floor for specimen design. The weights were evaluated considering the frame, steel frame for preventing collapse, steel jigs for measurement, and equipment for functional evaluation after an earthquake. Table 1 was used to design the allowable stress. Table 2 and Table 3 show lists of cross-sections of columns and beams. Note that the detail of the wall section can be found in reference [24,25].

The cross-sections of the columns were 500 mm squares, and the widths of the beams were all 300 mm. The length of the beam (G1) in the X-direction was 600 mm. The floor slab thickness was 130 mm. The concrete design strength *f*_d_ was set as 27 N/mm^2^. According to the pushover analysis, the base shear force coefficient at the maximum inter-story drift ratio of 0.01 rad (1%) was 0.35 in the X-direction.

The objective of this study was (1) the construction of a 2D model, which is often used in performance design, and (2) the evaluation of the accuracy of that model. The material test results are listed in Table 4 and Table 5 for reference. The concrete compressive strength *f*_c_ was 1.12–1.52 times the concrete design strength *f*_d_. For longitudinal and transverse reinforcement bars of column and beam, wall reinforcement bars, and slab reinforcement bars, the yield strength *f*_y_ was in the range of 370–957 N/mm^2^ and the ultimate strength *f*_u_ was in the range of 513–1055 N/mm^2^.

## 3. Overview of Shaking-Table Test Using Full-Scale Four-Story RC Building

### 3.1. Measurement

Experiments were conducted using E-Defense. A total of 679 channels were used to measure floor acceleration, inter-story drift, reinforcement bar strain, and others. Local deformations were measured, such as the shear deformation of the beam–column joint and wall and the vertical and horizontal displacement of the multistory wall. The damage of the columns, beams, column–beam joints, and walls were recorded by cameras. Data were measured at a sampling frequency of 200 Hz, but the data were resampled at 100 Hz for analysis. In this study, only the data measured with the servo-type accelerometer, the laser displacement sensor installed in each story, and the displacement transducer installed in the column–beam joint were used.

### 3.2. Input Seismic Motion and Experimental Schedule

In this experiment, the waves observed at the Japanese Meteorological Agency Kobe Local Meteorological Office (JMA-Kobe wave) and at the Japan Railway (JR) Takatori Station (JR-Takatori wave), which were recorded during the 1995 Hyogo-ken Nanbu Earthquake, were used. Table 6 lists the experimental schedule. The north–south (NS) component of the JMA-Kobe wave, which has a large maximum acceleration, was inputted in the Y-direction. The amplitude magnification of the JMA-Kobe wave in relation to the original wave was increased to 10%, 25%, 50%, and 100%. Subsequently, the JR-Takatori wave was used with the objective of confirming the situation of repeated large deformations and shaking-table tests with amplitude magnifications of 40% and 60% were conducted. White-noise-excitation experiments were also performed to identify the natural periods of the specimen building in each direction.

### 3.3. Summary of Experimental Results

Table 6 shows the maximum acceleration and maximum inter-story drift ratio measured in the experiment, and a natural period before and after the experiment. Although this paper is for the moment-resisting frame model, the experimental results of the seismic wall structure are also explained to provide information. In the X-direction, the natural period extended to 0.45 s after the 10% amplitude experiment, and 0.99 s after the 100% amplitude experiment, and 1.25 s after the 60% amplitude experiment of the JR-Takatori wave, which was the last experiment. In the Y-direction, the natural period was 0.34 s after the 10% amplitude experiment, 0.88 s after the 100% amplitude experiment, and 1.25 s after the last experiment. The maximum inter-story drift ratio was less than 0.003 rad (0.3%) along the X- and Y-directions up to the 25% amplitude experiment of the JMA-Kobe wave. The values were 0.016 rad (1.6%) and 0.0104 rad (1.04%) in the X- and Y-directions, respectively, in the 50% amplitude experiment of the JMA-Kobe wave. Herein, the values on the first and second floors were approximately twice as high as those of the third floor in the X-direction and almost equal on all floors in the Y-direction. The values were 0.034 rad (3.4%) and 0.033 rad (3.3%) in the X- and Y-directions, respectively, in the 100% amplitude experiment of the JMA-Kobe wave. The inter-story drift on the first and second floors significantly increased in the X-direction; the inter-story drift ratio on the first floor became relatively larger in the Y-direction. The values were 0.046 rad (4.6%) and 0.051 rad (5.1%) in the X- and Y-directions, respectively, in the 60% amplitude experiment in the final JR-Takatori wave.

**Figure 2:**
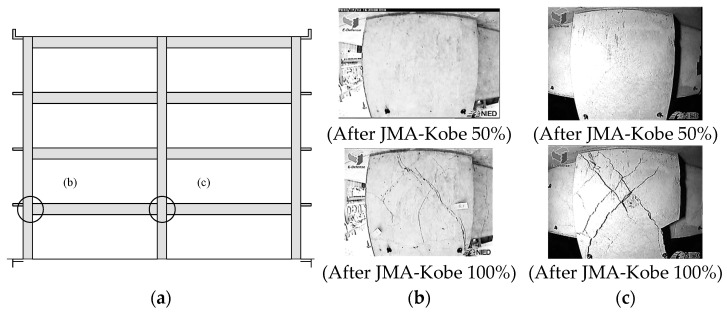
Beam–column damage observations: (**a**) recorded location; (**b**) damage of exterior beam–column joint after the JMA-Kobe tests with amplitudes 50% and 100%; and (**c**) damage of interior beam–column joint after the JMA-Kobe tests with amplitudes 50% and 100%.

The recorded locations of damage are shown in Figure 2a, and the beam–column joint damage after the experiments is shown in Figure 2b,c. In the 50% amplitude experiment of the JMA-Kobe wave, a large seismic motion caused cracks, but no severe damage was observed on the exterior or interior beam–column joint. However, the deformation of the joint was severe in the 100% amplitude experiment of the JMA-Kobe wave. In other words, the accuracy of the seismic performance of the 2D numerical model can be confirmed according to the joint damage using these experiment results. In this study, the inter-story drifts obtained from the experiment and analysis were also compared.

## 4. Analysis Method

The structural analysis program OpenSees [33] was used for analysis. The objective was to construct a 2D numerical model with the joints expressed as rigid regions, while the column and beam elements were described by a fiber model and were used to confirm the accuracy of the seismic response evaluation of the constructed 2D numerical model. Based on the objective of this study, detailed joint-modeling elements were not used even though the beam–column joint was damaged and the inter-story drift ratio was large, as mentioned in Section 3.3. The analyzed results of the natural period and mode shape were compared to improve the numerical model considering the effective slab width. The accuracy of the seismic performance evaluation of the model was quantitatively analyzed.

### 4.1. Analysis Modeling

**Figure 3:**
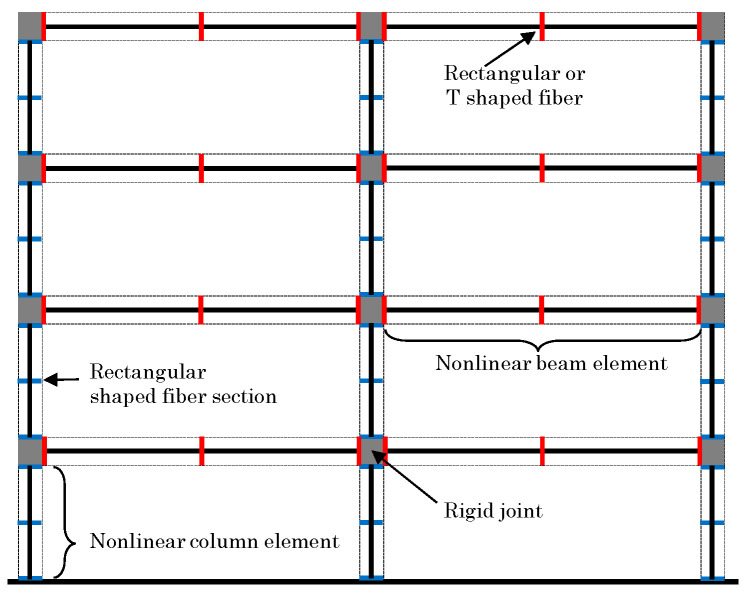
Numerical model with rigid joint and rectangular or T-shaped fiber sections for columns and beams.

The numerical model used in this study is shown in Figure 3. For the numerical model, one frame in the frame structure was considered. The floor of each model was set as a rigid diagram floor. Half of the weight of each floor shown in Table 1 was proportionally distributed to the area borne by the nodes.

**Figure 4:**
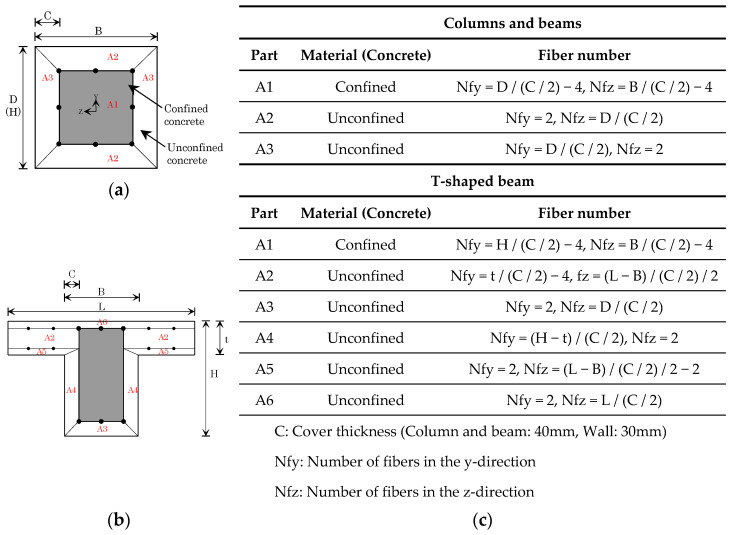
Modeling of structural elements: (**a**) columns and beams; (**b**) T-shaped beams; and (**c**) rules pertaining to fiber representations.

In this study, each member was modeled with a fiber model to construct a 2D numerical model wherein the cover thickness was divided into two parts and where each fiber was divided. Figure 4 shows a conceptual diagram of the fiber model of each member and the number of divisions (rounded integers) of each part.

**Figure 5:**
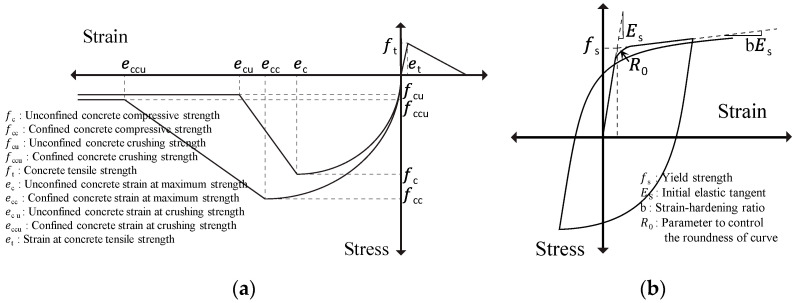
Constitutive material models in compression and in tension for (**a**) concrete; (**b**) steel.

The hysteresis models of concrete and reinforcement bars applied the Concrete02 [34,35,36] and Steel02 [37] material models, which were incorporated in OpenSees (Figure 5). Table 7 shows the parameters of the materials used in this study. The material property values were determined based on the current design code in Japan. The numerical model was generated with all members using the “nonlinearBeamColumn” element. The fiber model was divided based on the cross-sectional dimensions and reinforcement bar arrangement for the column and beam. The computed results differed according to the number of element divisions, but these were divided into three parts in reference to a previous study [38].

### 4.2. Analyzed Parameters

To confirm the effect of the effective slab width on the elastic analysis, a parametric analysis was conducted by generating effective slab widths from 0 mm up to 3600 mm at 25 mm intervals. Based on these results, a T-shaped beam cross-section was generated to consider the effective slab width in a seismic response analysis. Since the acceleration of the first floor was not measured in the 10% and 25% amplitude experiments of the JMA-Kobe wave, the seismic response analysis was conducted using the acceleration measured in the shaking-table test. The time step of the analysis was set to 0.01 s to reduce the analysis time, although the acceleration data of the shaking-table test were recorded with a sample rate of 200 Hz (0.005 s).

## 5. Comparison of Experimental and Analyzed Results

### 5.1. Natural Period and Modal Shape

**Figure 6:**
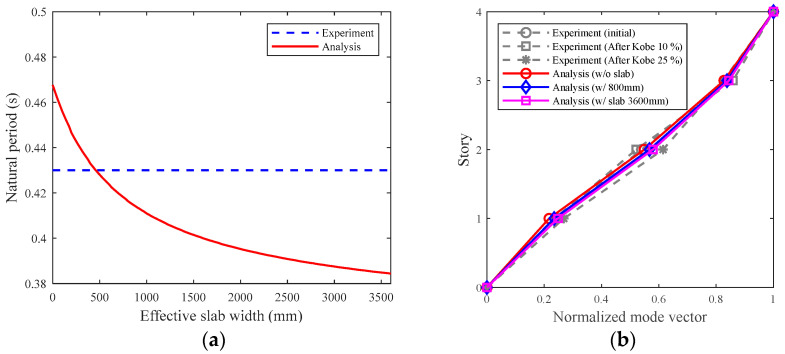
Results of eigenvalue analysis: (**a**) effects of effective slab width on the natural period; (**b**) effects of effective slab width on the first modal shape.

Figure 6a shows the result of the parametric analysis with the effective slab width at 25 mm intervals. The effective slab width of 3600 mm signifies that the total width of the slab of the specimen was considered. The natural period of the numerical model that did not consider the effective slab width was 0.47 s. When the effective slab width was considered to be 450 mm, the natural period obtained from the numerical model matched that obtained from the experiment. Figure 6b shows the modal shape obtained from the experiments, which were defined using the transfer function of each floor response acceleration of the specimen recorded in the white noise experiments, and that obtained from the eigenvalue analysis. The modal shape obtained from the experiments was the average of the modes obtained from the acceleration data of approximately 20 s in 10 sections during the white noise wave experiments. The modal shape obtained from the experiments corresponded with the mode shape obtained from the analysis. Additionally, there were few differences in the modal shape according to the effective slab width.

### 5.2. Floor Acceleration and Inter-Story Drift

**Figure 7:**
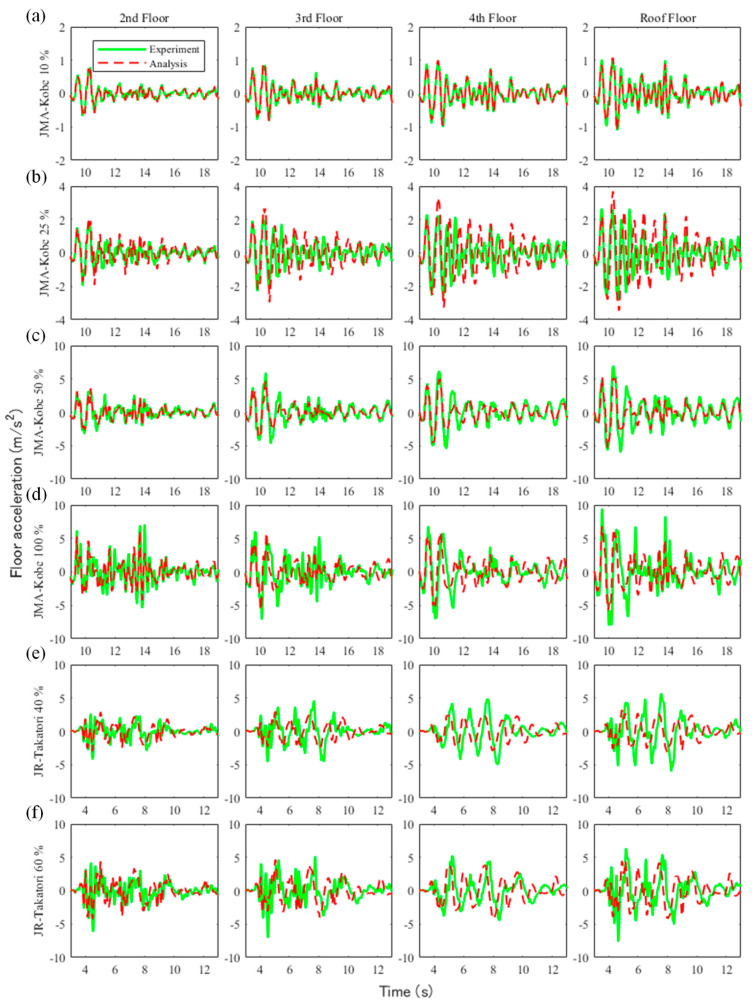
Comparison of the experimental and analyzed floor acceleration results: (**a**) JMA-Kobe 10% amplitude experiment, (**b**) JMA-Kobe 25% amplitude experiment, (**c**) JMA-Kobe 50% amplitude experiment, (**d**) JMA-Kobe 100% amplitude experiment, (**e**) JR-Takatori 40% amplitude experiment, and (**f**) JR-Takatori 60% amplitude experiment.

**Figure 8:**
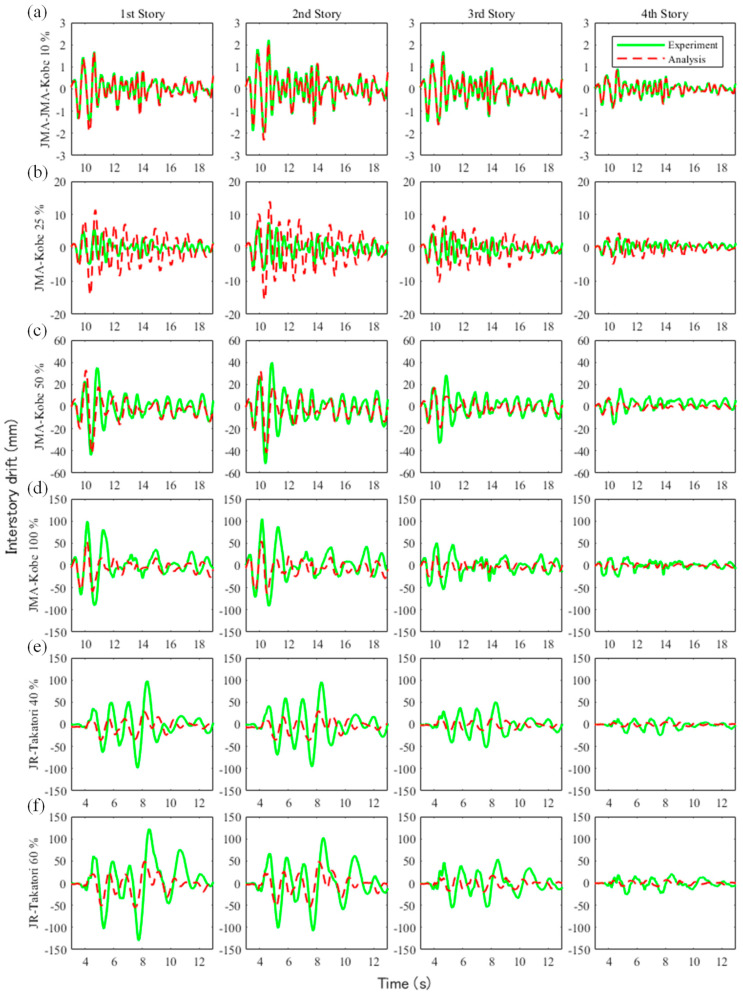
Comparison of the experimental and analyzed inter-story drift results: (**a**) JMA-Kobe 10% amplitude experiment, (**b**) JMA-Kobe 25% amplitude experiment, (**c**) JMA-Kobe 50% amplitude experiment, (**d**) JMA-Kobe 100% amplitude experiment, (**e**) JR-Takatori 40% amplitude experiment, and (**f**) JR-Takatori 60% amplitude experiment.

Figure 7 and Figure 8 show the floor acceleration and inter-story drift obtained from the experiments and analysis. In the figure, the solid green line denotes the experimental results, and the red dotted line is the analysis results. Table 8 shows the root-mean-square percentage error (RMSPE) of the maximum acceleration and maximum inter-story drift ratio between the experimental and analytical results obtained from the following equation:

(1) $$\mathrm{RMSPE}=\sqrt{\frac{1}{n}\sum_{k=1}^{n}{\left(\frac{Xmsed_{k}-Xanal_{k}}{Xmsed_{k}}\right)}^{2}},$$

where Xmsed is the measured value and Xanal is the value obtained from the analysis. In the 10% amplitude experiment of the JMA-Kobe wave, as shown in Figure 7a and Figure 8a, the floor acceleration and inter-story drift of the analysis were in good agreement with the experimental results. Therefore, the RMSPEs were 0.04% for the floor acceleration and 0.07% for the inter-story drift. In the 25% amplitude experiment of the JMA-Kobe wave, as shown in Figure 7b and Figure 8b, the floor accelerations obtained from analyses were in good agreement with the lower floors, but the accuracy decreased on higher floors. The same tendency was observed in the inter-story drift. Therefore, the RMSPE was larger than the 10% amplitude experiment of the JMA-Kobe wave. In the 50% amplitude experiment of the JMA-Kobe wave, as shown in Figure 7c and Figure 8c, the floor acceleration obtained from the analyses was in good agreement for all floors. However, the inter-story drift accuracy decreased on higher floors. The RMSPE in the 50% amplitude experiment of the JMA-Kobe wave was 1.03% for the floor acceleration and 4.7% for the inter-story drift. However, the difference value was small in terms of the seismic performance evaluation. Using the measured acceleration on the first floor in the 50% amplitude experiment of the JMA-Kobe wave, the maximum acceleration and maximum velocity were 3.75 m/s^2^ and 36.15 cm/s, respectively. This motion corresponded to a seismic motion that slightly exceeded VII on the modified Mercalli intensity (MMI) scale, which is extensively used worldwide [39]. In other words, in terms of the seismic performance evaluation, it could be inferred that the analytical model used in this study has sufficient accuracy to evaluate the seismic performance of buildings constructed in regions with a maximum seismic intensity of VII. In the 100% amplitude experiment of the JMA-Kobe wave and the 40% and 60% amplitude experiments of the JR-Takatori wave, as shown in Figure 7d,e and Figure 8d,e, the accuracy of the floor acceleration and inter-story drift obtained from the analysis decreased. The inter-story drifts on the lower floors in particular were smaller than the experimental results. Because the beam–column joint was modeled as a rigid member in this study, its deformations were not able to be evaluated. Therefore, it is desirable to evaluate seismic performance using a numerical model with nonlinear behavior of the beam–column joint in areas with a maximum seismic intensity of VIII (or larger).

### 5.3. Seismic Performance Curve

**Figure 9:**
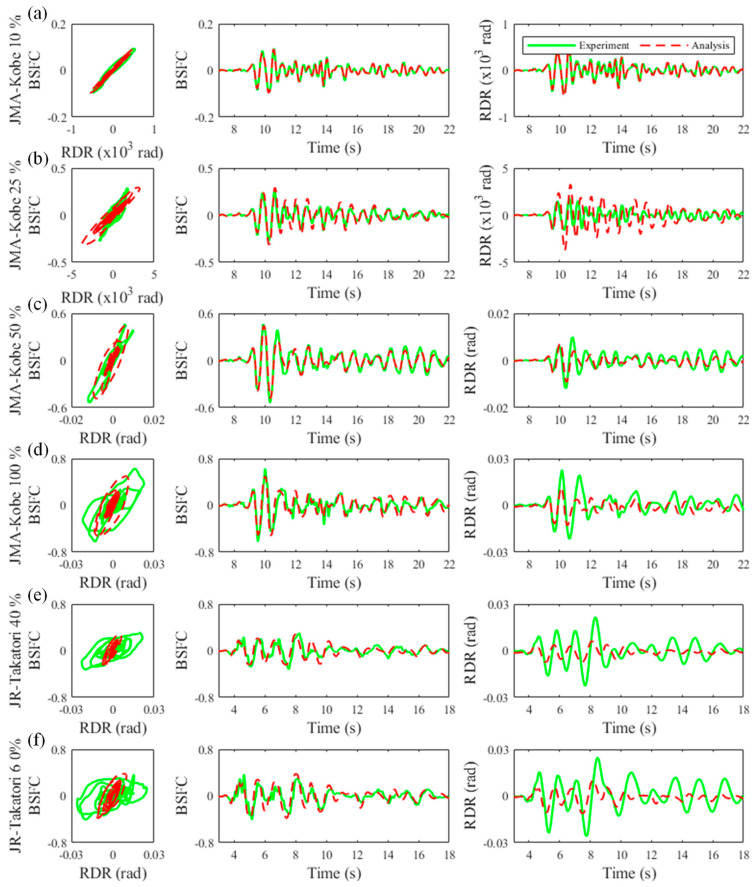
Comparison between hysteresis loops of base shear force coefficient (BSFC) and roof drift ratio (RDR) obtained from experiments and analyses: (**a**) JMA-Kobe 10% amplitude experiment, (**b**) JMA-Kobe 25% amplitude experiment, (**c**) JMA-Kobe 50% amplitude experiment, (**d**) JMA-Kobe 100% amplitude experiment, (**e**) JR-Takatori 40% amplitude experiment, and (**f**) JR-Takatori 60% amplitude experiment.

Figure 9 shows the relationship between the base shear force coefficient that was defined using each floor acceleration and weight and the roof drift ratio that was defined using the roof displacement and building height, hereafter referred to as the seismic performance curve. In the 10% amplitude experiment of the JMA-Kobe wave, as shown in Figure 9a, the seismic performance curves obtained from the analysis and experiment were in good agreement.

However, in the 25% amplitude experiment of the JMA-Kobe wave, as shown in Figure 9b, the seismic performance curve obtained from the analysis was not close to that obtained from the experiment. This was thought to be caused by the inter-story drift obtained from the analysis being larger than the experimental results, as shown in Figure 8b. In the 50% amplitude experiment of the JMA-Kobe wave, as shown in Figure 9c, the seismic performance curve obtained from the analysis was comparable to the experimental results. In the 100% amplitude experiment of the JMA-Kobe wave, as shown in Figure 9d, the analyzed results were comparable to the experimental results to some extent in the first part of the experiment; however, the accuracy decreased because the roof drift ratio obtained from the analysis was small after approximately 10 s. In the 40% and 60% amplitude experiments of the JR-Takatori wave, as shown in Figure 9e,f, the drop in accuracy of the seismic performance curve obtained from the analysis implied that the joint deformation was not considered.

### 5.4. Maximum Inter-Story Drift Ratio

**Figure 10:**
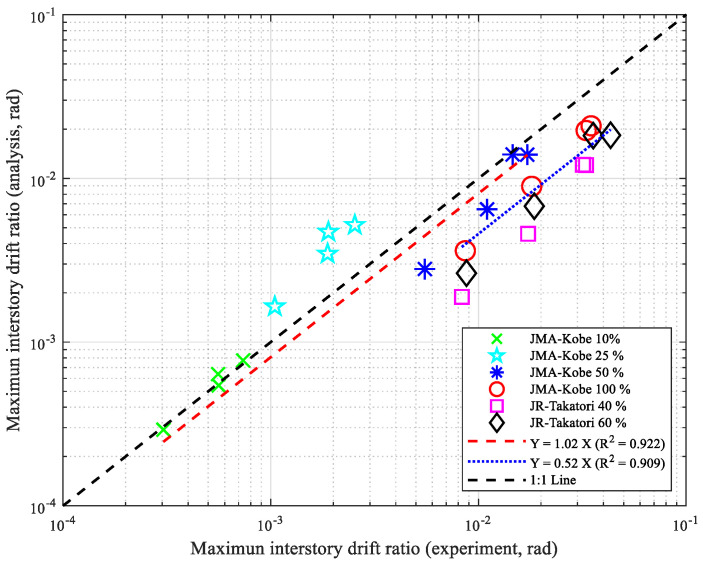
Comparison between maximum inter-story drift ratio obtained from experiments and analyses on log–log plot.

Figure 10 shows the maximum inter-story drift ratio obtained from analysis and experiments. The horizontal axis of the figure denotes the experimental results, and the vertical axis shows the analyzed results. In the 10–50% amplitude experiments of the JMA-Kobe wave, the values were plotted around the 1:1 line, even though the 25% amplitude experiment results were plotted above the 1:1 line (black dotted line). In the 100% amplitude experiment of the JMA-Kobe wave and the 40% and 60% amplitude experiments of the JR-Takatori wave, the values were plotted below the 1:1 line. A regression analysis was performed for the two groups of values based on the 50% amplitude experiment of the JMA-Kobe wave. In Figure 10, the red dotted line is the regression of the experimental and analyzed results of the 10–50% amplitude experiments of the JMA-Kobe wave; this had a relationship of 1:1.02, which showed that the analyzed results corresponded well with the experimental results. In Figure 10, the blue dotted line is the regression of the experimental and analyzed results of the 100% amplitude experiment of the JMA-Kobe wave and the 40% and 60% amplitude experiment of the JR-Takatori wave; the relationship was 1:0.51, which showed that the accuracy of the correspondence between the analytical and experimental results was poor. Since the peak maximum inter-story drift ratio obtained with the 50% amplitude experiments of the JMA-Kobe wave was 0.016 rad (1.6%), it can be inferred that the seismic performance was accurately estimated when the peak maximum inter-story drift ratio of the analyzed results in the numerical model was less than 0.015 rad (1.5%).

## 6. Conclusions

In evaluating the seismic response of buildings in moderate seismic regions, the objective of this study was the discussion of a 2D numerical model of a frame structure. This study was based on a four-story RC building experiment conducted in 2010 at a full-scale earthquake testing facility commonly referred to as “E-Defense”. In this study, the 2D numerical model was composed of a square fiber section model for the columns, T-shape fiber section model for the beam and slab, and rigid joint model for the beam–column joint. The accuracy of the seismic performance evaluation of the 2D numerical model was quantitatively evaluated based on the analyzed results. The findings obtained in this study are summarized below.

1. When using the 2D numerical model that applied the material property values used in the current designs in Japan, the natural period of the 2D numerical model considering the effective slab width of 450 mm was close to that obtained from the first white-noise-excitation experiment. It was also shown that the elastic modal shape of the structure could be estimated from this analysis using the 2D numerical model.
2. In the 50% amplitude experiment of the JMA-Kobe wave, the root-mean-square percentage errors of the 2D numerical model investigated in this study were 1.03% for the floor acceleration and 4.7% for the inter-story drift. In other words, it was confirmed that this model had sufficient accuracy in evaluating the seismic performance of buildings in regions whose maximum seismic motion was VII on the modified Mercalli intensity (MMI) scale, which is close to the 50% amplitude experiment of the JMA-Kobe wave.
3. The outcomes of the regression analysis of the peak maximum inter-story drift ratio obtained from the experiments and analyses for the experiments in the range of 10–50% of the JMA-Kobe wave had a relationship of 1:1.02, indicating a good correspondence between the analyzed and experimental results. The regression results of the peak maximum inter-story drift ratio for the experiments in excess of 50% of the JMA-Kobe wave had a relationship of 1:0.51. In other words, this model could accurately estimate the seismic performance in conditions wherein the peak maximum inter-story drift ratio of the analyzed results was less than 0.015 rad (1.5%).

To be used for the performance evaluation of the seismic frame of a building constructed in an area wherein moderate seismic motions frequently occur, the 2D numerical model with the rigid joint model was investigated in this study. For the evaluation of the seismic response of a frame building with non-seismic joint details or a seismic-resisting frame constructed in a strong seismic area, a nonlinear behavior model for beam–column joint is needed. This will be a topic for future study.

## Figures and Tables

**Table 1:** Floor weights.

Floor	Structural Elements [kN]	Nonstructural Elements [kN]	Total Weight [kN]
roof floor	816	118	934
fourth floor	853	14	867
third floor	849	23	872
second floor	845	23	867

**Table 2:** Cross-sections and reinforcement details of columns.

Floor	Size		C1		C2
4 FL~3FL	cross-section		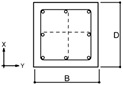		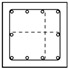
	dimensions (B × D)		500 × 500		500 × 500
	longitudinal bar		8–D 22		10–D 22
	transverse reinforcement	joint	[2,2] D10@140		[2,2] D10@140
		column	[2,2] D10@100		[2,2] D10@100
2 FL	cross-section		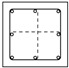		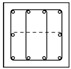
	dimensions (B × D)		500 × 500		500 × 500
	longitudinal reinforcement		8–D 22		10–D 22
	transverse reinforcement	joint	[2,2] D10@140		[2,2] D10@140
		column	[2,2] D10@100		[2,2] D10@100
1 FL	cross-section		Top	Bottom	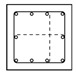
			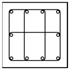	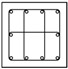	
	dimensions (B × D)		500 × 500	500 × 500	500 × 500
	longitudinal reinforcement		8–D 22	8–D 22	10–D 22
	transverse reinforcement	joint	[2,2] D10@140	[2,2] D10@140	[2,2] D10@140
		column	[2,2] D10@100	[2,2] D10@100	[2,2] D10@100

**Table 3:** Cross-sections and reinforcement details of exterior and interior beams.

Properties		Floor	Beam (G1)			Floor	Beam (G1)		
section	location	RFL	outer	center	inner	3FL	outer	center	inner
	cross-section		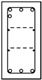	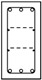	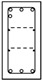		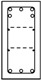	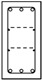	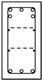
dimension (B × D)			300 × 600				300 × 600		
longitudinal reinforcement	top		4–D22	3–D22	4–D22		5–D22	3–D22	5–D22
	bottom		3–D22	3–D22	3–D22		3–D22	3–D22	3–D22
transverse reinforcement			2-D10@200				2-D10@200		
section	location	4FL	outer	center	inner	2FL	Outer	Center	Inner
	cross-section		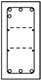	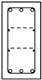	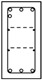		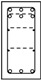	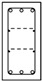	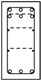
dimension (B × D)			300 × 600				300 × 600		
longitudinal reinforcement	top		4–D22	3–D22	4–D22		6–D22	3–D22	6–D22
	bottom		3–D22	3–D22	3–D22		3–D22	3–D22	3–D22
transverse reinforcement			2-D10@200				2-D10@200		

**Table 4:** Material properties of concrete (Unit: MPa).

Structural Elements	*f*_d_	*f*_c_
bottom part of column of fourth story and roof floor slab	27	41
bottom part of column of third story and fourth floor slab	27	30.2
bottom part of column of second story and third floor slab	27	39.2
bottom part of column of first story and second floor slab	27	39.6

**Table 5:** Material properties of reinforcement bar (Unit: MPa).

Label	Grade	Structural Elements	*f*_y_	*f*_u_
D22	SD345	longitudinal reinforcements of columns and beams	370	555
D19	SD345	longitudinal reinforcements of beams	380	563
D13	SD295	vertical reinforcements of walls	372	522
D10	SD295	and horizontal reinforcements of walls and transverse reinforcements of columns and beams	388	513
D10	SD295	welded hoop for transverse reinforcements of columns and beams	448	545
D10	KSS785	transverse reinforcements of beams	952	1055

**Table 6:** Maximum accelerations, maximum inter-story drift ratios, and periods [25].

Test No.	Input Wave	Maximum Acceleration (m/s^2^)			Maximum Inter-Story Drift Ratio (rad)		Period (s)	
		X-Dir.	Y-Dir.	Z-Dir.	X-Dir.	Y-Dir.	X-Dir.	Y-Dir.
1	JMA-Kobe 10%	0.69	0.98	0.35	0.0005	0.0006	0.45	0.34
2	JMA-Kobe 25%	1.66	2.69	0.96	0.0021	0.0027	0.47	0.37
3	JMA-Kobe 50%	3.49	4.66	1.98	0.016	0.0104	0.66	0.57
4	JMA-Kobe 100%	7.88	10.67	4.15	0.0343	0.034	0.99	0.88
5	JR-Takatori 40%	3.05	3.34	1.69	0.0342	0.0269	1.13	1.02
6	JR-Takatori 60%	4.54	5.46	1.69	0.0457	0.0551	1.25	1.25

**Table 7:** Modeling material parameters for structural elements.

**(a) Concrete**		
**Parameter**	**Unconfined**	**Confined**
$f_{c}\left(f_{cc}\right)$	−27 MPa	$f_{cc}=f_{c}\times \kappa$ [17]
$e_{c}\left(e_{cc}\right)$	−0.002	−0.003
$f_{cu}\left(f_{ccu}\right)$	$f_{c}\times 0.2$	$f_{cc}\times 0.2$
$e_{cu}\left(e_{ccu}\right)$	−0.004	$e_{cc}\times 20$
$f_{t}$	$33,500\times k_{1}\times k_{2}\times {\left(\gamma /24\right)}^{1/2}\times {\left(f_{c}/60\right)}^{1/3}$ [5]	
$E_{c}$	$0.33\times {\left(f_{c}\right)}^{1/2}$ [32]	
**(b) Steel**		
$f_{s}$	345 MPa (SD345), 295 MPa (SD295)	
$E_{s}$	205,000 MPa	
b	0.01	
$R_{0}$	18	

**Table 8:** Root-mean-square percentage error for maximum floor acceleration and maximum inter-story drift ratio (experimental and analyzed results, unit: %).

Test No.	Input Wave	Maximum Floor Acceleration	Maximum Inter-Story Drift Ratio
1	JMA-Kobe 10%	0.04	0.07
2	JMA-Kobe 25%	0.51	2.09
3	JMA-Kobe 50%	1.03	4.7
4	JMA-Kobe 100%	1.02	12.76
5	JR-Takatori 40%	1.02	15.02
6	JR-Takatori 60%	1.22	21

## Data Availability

Not applicable.
